# Editorial: Toward Consumer 4.0 Insights and Opportunities Under the Marketing 4.0 Scenario

**DOI:** 10.3389/fpsyg.2020.611114

**Published:** 2021-01-11

**Authors:** María Pilar Martínez-Ruiz, Mónica Gómez-Suárez, Ana Isabel Jiménez-Zarco, Alicia Izquierdo-Yusta

**Affiliations:** ^1^Department of Business Administration, University of Castilla-La Mancha, Albacete, Spain; ^2^Department of Finance and Marketing Research, Autónoma University of Madrid, Madrid, Spain; ^3^Department of Economic and Business, Open University of Catalonia, Barcelona, Spain; ^4^Departament of Economic and Business Administration, University of Burgos, Burgos, Spain

**Keywords:** marketing 4.0, consumer 4.0, technology, virtual, COVID

This Research Topic aims to shed light on the current academic and practical perspectives within the so-called “Consumer 4.0” paradigm. The related perspective on Marketing 4.0 represents new opportunities and concerns for Social Science. Thus, there is a need to find a balance between theoretical frameworks, opinions, and empirical applications, and thereby stimulate dialogues among researchers and professionals. In short, this collection of papers encapsulates the literature on how technological evolution is impacting different actors (companies, consumers, patients, and tourists, etc.) and interacting in various sectors. We also propose research lines based on the combination of two key factors: the advancement of technologies and the impact of the SARS-COV-2 pandemic.

This collection builds upon a previous Research Topic, “From consumer experience to affective loyalty: challenges and prospects in the psychology of consumer behavior 3.0,” which influenced subsequent research on the opportunities and challenges inherent to Marketing 4.0. The primary objective of the current Research Topic is to analyze how virtual transformation affects the behavior of very different organizations, industries, and consumers.

This Research Topic collection includes 15 research articles spanning diverse publication formats, including 8 Original Research Articles, 4 Opinion Reviews, and 3 Mini-reviews. Although different, the papers have common threads, examining the impact of Information and Communication Technology (ICT) on market demand and business strategy. They are also connected either through the functional scope within the organization or the focal sector in which they are applied.

Blazquez-Resino et al. highlight how for companies to be competitive, it is important to establish synergy between Industry 4.0 and Marketing 4.0. Muñoz et al. discuss how investments in technology influence the orientation toward entrepreneurship in SMEs, showing how digital transformation has allowed direct and rapid access to information about consumers in an atomized market, in this case, wine. In an examination of the financial field, Callejas-Albiñana et al. conducted a panel data study among a selected sample of countries to determine the influence of different exogenous factors, aiming to explain stagnation in recent years and outline proposals for new government intervention strategies.

In Marketing 4.0, which reflects the impact of the technological revolution on the marketing arena (Jiménez-Zarco et al., 2019), companies face high volumes of information (Big Data) (Lies, 2019) that can be analyzed through data mining techniques (Data Mining) (Sener et al., 2019).

Studies on “Big Data” illustrate the importance of capturing and analyzing huge amounts of information and Rubio et al. underscore the importance of virtual communities on the co-creation process. Díaz-Martín et al. analyzed the concept of health e-mavens, showing the importance of conducting investigations with data mining and user-generated content. Through an in-depth review of financial literature, Díaz and Esparcia analyzed investors' risk aversion in terms of different environments, financial products, and temporality, etc. The authors utilized Big Data on financial products to uncover new research lines for risk reduction.

Virtual developments also allow scholars to better measure the impact of business strategies on an individual's behavior, namely by monitoring activities in cyberspace, which include search engine queries, social network relationships, and website purchases. In this regard, Wang et al. used behavioral and ERP (event-related potentials) measures to explore the priming effects of monetary and social rewards on e-commerce consumer decisions in China. In their analysis of individual behavior in relation to specific advertising strategies, García-Madariaga et al. reveal the importance of using neurophysiological measures to analyze the appropriate use of visual metaphors, as well as how to maximize impact and ensure that advertising is effective.

The extensive use and adoption of technology has not only impacted organizations, it has given rise to a new type of consumer: Consumer 4.0. In an omni-channel context, combining digital and physical media, this type of customer maintains positive and lasting relationships with multiple firms and other actors. However, the characteristics of digital media are particularly impactful on these individual's brand decisions as they complete their brand journey. In this vein, Martinez-Ruiz and Moser analyzed the evolution of the worldwide web and its impact on consumer behavior.

As the online environment has evolved, scholars have emphasized the importance of the psychological aspects of consumer behaviour—namely, individual preferences, emotions, and sensory experiences. For instance, Bettiga and Lamberti shed light on the still vague concepts of anticipated and anticipatory happiness. Their results demonstrate the importance of designing visual product communication in a way that elicits positive feelings of anticipated and anticipatory emotions in the viewer. Building upon gratifications theory, Rodriguez-Ardura and Messeguer-Artola developed an integrative and context-specific model that links engagement with enjoyment, self-presentation, and community belonging, which were all identified as motivational factors among Facebook users. Meanwhile, Reinares-Lara et al. measured the experience of people through two dimensions of satisfaction: cognitive (the most studied so far in academic research) and affective (the least analyzed), derived from the analysis of neurophysiological data.

According to the previously mentioned category, the tourism industry provides useful case studies for understanding the huge impact of technology. Blazquez-Resino et al. show how ICTs are important for understanding the development of loyalty, making an important distinction between passive attitudinal loyalty, active attitudinal loyalty, and behavioral loyalty. Huete-Alcocer et al. discuss how building an image of a holiday destination is a critical factor in the perceptions and evaluations of that destination by tourists. This study accounts for not only cognitive and affective components but also the unique image component. Moreover, they demonstrate that eWOM is a powerful means of promoting cultural tourism. Focusing on shopping tourism, Muro-Rodríguez et al. highlight its importance for cities, identifying differentiation strategies for cities as shopping destinations and setting out recommendations based on the analysis of key factors for listing a city as a shopping destination.

## Future Research Directions

Since the start of the SARS-COV-2 pandemic in early 2020, consumers and companies have faced a new environmental variable that has accelerated the process of digitization and the incorporation of new technologies. This exogenous phenomenon will produce changes in the global economy, organizational management, and consumer habits. Some will be transitory, while others will be permanent. Future papers (and topics) therefore need to investigate how the pandemic will interact with Marketing 4.0 and influence the competitive strategies that allow organizations to obtain competitive advantages and offer adapted products and services to meet these new demands. Notably, disruptive or emerging technologies that companies were previously using as a test have been incorporated in a masive scale into infrastructures in many countries during the pandemic.

For example, traditionally, neuroscientific techniques have been used to measure consumer reactions to advertising-related stimuli or to assess the effectiveness of discount coupons or gifts. Easy-to-use neuro-marketing tools are now being implemented to measure the emotions and experiences of consumers from neurophysiological data relating to a wide range of companies from different sectors (including retail, health, and tourism, etc.) and/or diverse purchasing contexts (such as online, offline, and omnichannel, etc.).

Scholars with access to longitudinal data (pre- and post- COVID-19), should measure buyer behavior in terms of the variables that influence them before, during, and after the decision-making process.

These variables include the impact of perceived risk, which leads consumers to conduct a more systematic and prolonged process of searching for information, mainly on social networks or web pages (Hansen et al., 2018). The relationship between its different components (physical, functional, economic, social, and psychological) may also change based on the product category (Emilien et al., 2017). However, the economic crisis inflicted by the pandemic may produce the opposite effect. Even though the perceived risk is high, certain middle-class segments that were previously financially healthy might be struggling with their basic food shopping.

The next variable relates to consumer preferences for different attributes/values and whether they have remained stable or changed. In the mass consumer sector, and particularly in food or personal hygiene, it is worth studying whether consumers are more likely to use safety as a criterion for choosing a product or not. Following on from this, in the future, scholars should explore technological ways of tracking or tracing health or food products.

## Author Contributions

All authors listed have made a substantial, direct and intellectual contribution to the work, and approved it for publication.

## Conflict of Interest

The authors declare that the research was conducted in the absence of any commercial or financial relationships that could be construed as a potential conflict of interest.
